# Ambient temperature extremes and neonatal mortality: a time-stratified case-crossover analysis using Demographic and Health Survey data from East Africa (2011–2022)

**DOI:** 10.1136/bmjph-2025-004085

**Published:** 2026-07-22

**Authors:** Paul Lokubal, Chérie Part, Sari Kovats, Debra Jackson, Hannah K Blencowe, Chloe Brimicombe, Veronique Filippi

**Affiliations:** 1 Department of Infectious Diseases and International Health, Faculty of Epidemiology and Population Health, London School of Hygiene & Tropical Medicine, London, UK; 2 Department of Public Health, Environments and Society, Faculty of Public Health and Policy, London School of Hygiene & Tropical Medicine, London, UK

**Keywords:** Public Health, Environmental Exposure, methods

## Abstract

**Introduction:**

Due to their underdeveloped thermoregulatory system, neonates are at increased risk of morbidity and mortality from hot and cold temperatures. Our study aimed to analyse the effects of environmental temperature on overall, very early, early and late neonatal acute mortality in five East African countries using the Demographic and Health Surveys (DHS) data.

**Methods:**

We obtained neonatal mortality data from the DHS conducted between 2016 and 2022, capturing births and deaths occurring between 2011 and 2022. Our outcomes were (1) overall neonatal mortality (days 0–27), (2) very early (day 0); (3) early (days 1–6) and (4) late neonatal mortality (days 7–27). Daily mean temperature was constructed from ERA5-Land and assigned at the household level. We used a time-stratified case-crossover design with distributed lag non-linear models (0–6-day lag) to estimate odds of mortality with exposure to the 5th and 95th temperature percentiles (vs the median). Country-level estimates were generated and then pooled to assess the overall association.

**Results:**

A total of 1373 neonatal deaths were included, over 80% of which occurred within the first 6 days of life. The association between ambient temperature and neonatal mortality was heterogeneous. In pooled analysis, the 95th and 5th percentiles were associated with increased and decreased mortality odds respectively, although estimates were imprecise. In Uganda, there was strong evidence of association between high ambient temperature (95th percentile) and overall neonatal mortality (OR=3.54; 95% CI 1.73 to 7.28) as well as early neonatal mortality (OR=3.75; 1.70 to 8.28), while odds of very early neonatal mortality increased with exposure to low temperatures (5th percentile) (OR=5.65; 1.89 to 16.69). There was no strong evidence of association in other countries.

**Conclusion:**

Temperature-related neonatal mortality risk differs across East African countries. Other factors may play a significant role. Future research should consider the effects of environmental temperature on neonatal mortality across different climate zones.

**WHAT IS ALREADY KNOWN ON THIS TOPIC**

- Hot and cold indoor environments are associated with an increased risk of neonatal mortality.
- Neonatal vulnerability to temperature extremes is most pronounced in the early neonatal period (days 0–6) compared with the late neonatal period (days 7–27).

**WHAT THIS STUDY ADDS**

- This study adds to the body of evidence on the effect of heat on neonatal mortality in different climate regions, allowing for between-country assessment of the association between temperature and neonatal mortality in East Africa.
- Our analysis provides country-level estimates for the association between temperature and overall neonatal mortality, as well as the three distinct phases of the neonatal period (very early, early and late) with differing mortality risk from temperature.

**HOW THIS STUDY MIGHT AFFECT RESEARCH, PRACTICE OR POLICY**

- There is a need to incorporate heat mitigation strategies into the care for newborn policies and practices, especially for the very early and early neonatal periods.

## Introduction

 Childhood mortality remains an important global health challenge. The neonatal period, defined as the first 28 days of postnatal life, represents a critical phase for child survival, particularly in low- and middle-income countries (LMICs).^1,2^ While significant progress has been made in reducing childhood mortality since 1990, the decline in neonatal mortality has been slower than under-5 (U5) mortality.^1,2^ For example, between 2000 and 2023, the global U5 mortality rate decreased by 52% while the neonatal mortality rate (NMR) decreased by 44%.^2^

Nearly 50% of all U5 deaths in 2023 were in the neonatal period.^2^ In 2023 alone, approximately 2.3 (2.1–2.6) million newborns died within the neonatal period, with the majority occurring in LMICs.^1^ Sub-Saharan Africa (SSA) has the highest NMR of 27 deaths per 1000 live births in 2023, compared with 17 per 1000 globally and two deaths per 1000 live births in Europe, Australia and New Zealand.^2^ In East Africa, NMR remains high and varies between 18 and 22 deaths per 1000 live births, with Kenya and Tanzania having the highest rates at 21.5 (17.4–26.4) and 20.6 (15.6–27.0), respectively, followed by Burundi (19.6, 11.3–34), Rwanda (18.1, 13.5–24.1) and Uganda (17.9, 11.2–27.8).^2^ Moreover, at the current pace of decrease in NMR at 34%, the whole of SSA, except South Africa, is at risk of missing the Sustainable Development Goal NMR target of ≤12/1000 live births by 2030.^2^ The early timing of most newborn deaths implies a narrow window for life-saving interventions, with approximately 33% occurring on the day of birth and 75% within the first week.^1,3^

The leading medical causes for neonatal mortality include prematurity, birth complications (birth asphyxia), congenital anomalies, lower respiratory infections (pneumonia) and sepsis, which account for 86% of all neonatal mortalities worldwide.^1,2,4^ Recent research has also demonstrated the significant impact of environmental factors, such as air pollution and temperature, on neonatal mortality.^5–8^ Extreme heat events are becoming more frequent in Africa.^9,10^ One study analysed data from 29 countries and found that, over an 18-year period (2001–2019), 4.3% of neonatal deaths were associated with non-optimal temperatures, with heat and cold responsible for 1.5% and 2.9% of all neonatal deaths, respectively.^5^ However, this study pooled data from all 29 countries, obscuring between- and within-country temperature differences and its effects on neonatal mortality. Nevertheless, these findings highlight the important role of non-optimal temperatures on neonatal mortality.

Hypothermia remains high and variable among newborns, ranging 32%–85% among those born in hospitals and from 11% to 92% among those born at home, even in tropical climates.^11^ A Norwegian observational study explored the normal temperature range and risk factors for deviating body temperature during the first 24 hours of life in term-born infants under standardised care. They showed that, even when delivery room and rooming-in temperatures were maintained at the optimal 26°C–30°C and 24°C, respectively, 28% of infants still experienced hypothermia.^12^ These findings highlight the narrow ambient temperature range needed to maintain neonatal core body temperature at an optimal level. Conversely, exposure to high ambient temperatures poses equally serious risks: neonatal hyperthermia can result in dehydration, heat stress, respiratory distress and increased susceptibility to infections.^13,14^ A systematic review and meta-analysis across 66 countries found increased odds of preterm birth of 1.04 per 1°C increase in temperature and 1.26 during heatwaves,^7^ with wide-ranging maternal, fetal and neonatal adverse health effects.

Neonates are susceptible to environmental temperatures due to their underdeveloped thermoregulatory systems.^15,16^ Their high surface area to volume ratio facilitates rapid heat loss or gain, while limited subcutaneous fat, especially in preterm infants, reduces their ability to maintain core body temperature.^3,15^ The underdeveloped thermoregulatory centre in the hypothalamus, inefficient shivering mechanism and underdeveloped sweat glands further compromise neonates’ ability to regulate body temperature.^3,17^ This vulnerability is most pronounced in the very early (0 to <24 hours post partum) and early neonatal (1–6 days post partum) periods when newborns are adapting to the extrauterine environment.^15^ There is also evidence that daily temperature extremes exacerbate several causes for neonatal mortality, including respiratory distress and exposure to environmental pathogens, as well as increase susceptibility to infections, further contributing to neonatal mortality.^17,18^ This risk is even greater for preterm infants and those with low birth weight, who face greater challenges in temperature regulation.^3,12,19^ In LMICs, additional factors such as inadequate healthcare facilities, lack of temperature-controlled environments and socioeconomic constraints further exacerbate these risks.^5,20^

Despite growing recognition of the impacts of ambient temperature on neonatal health, there remains a significant gap in understanding how these effects manifest across the very early, early and late neonatal periods. First, these periods present distinct health challenges to the newborn, as outlined above.^1,3^ To our knowledge, only one study has explored temperature’s differential impact on neonatal mortality, but considered only the overall neonatal (0–28 days) and very early neonatal (day 0) periods,^5^ omitting the distinct early and late neonatal periods. Second, the few existing cross-country studies assessing the effects of temperature on neonatal health outcomes have typically pooled data across countries, which does not allow for between-country differences in the exposure–response relationships.^5,21^ SSA is a large continent with different climate zones; therefore, pooling results across them could obscure important regional differences. For example, compared with West Africa, there is less evidence of heatwaves in East Africa, although some countries in the region,^22^ including Uganda and Kenya, are projected to experience some of the greatest increases in the number of hot days.^23^ The five countries included in this study experience intra-annual temperature ranges from 11.6°C to 30.7°C, with lower temperatures at high altitudes and higher temperatures in lowland and coastal regions.^24^ Kenya and Tanzania, which lie along the coast, have the most diverse temperature ranges from 10°C to 34°C and 10°C to 32°C, respectively. Uganda averages 17°C–25°C, while Rwanda and Burundi have milder temperatures, ranging between 10°C–27°C and 16°C–23°C, respectively.^24^ Our study set out to: (1) explore the impact of ambient temperature extremes on overall, very early, early and late neonatal mortality at country-level; and (2) compare the effects of ambient temperature extremes on the pooled overall, very early, early and late neonatal mortality in five East African countries using the latest Demographic and Health Survey (DHS) data.

## Methods

### Study population

The DHS are nationally representative household surveys conducted in overlapping 5-year phases in over 90 LMICs since 1984.^25^ They provide important maternal, newborn and child health information. We conducted a secondary analysis of DHS datasets from five East African countries (Uganda, Kenya, Tanzania, Rwanda and Burundi), conducted between 2016 and 2022, capturing births and deaths occurring between 2011 and 2022.^25–27^ We chose the most recent DHS because they include the reported date of birth and date of death for newborns dying within the first 28 days of life,^25–27^ which are needed to accurately assign exposures. Women aged 15–49 years were interviewed about their complete birth and child health histories for all the births in the 5 years preceding the survey. Sampling follows a stratified two-stage cluster design: the first stage involved enumeration areas (EAs), also known as primary sampling units (PSUs), which are usually drawn from national census files using probability proportional to size and the second stage involves random sampling of households listed under each EA.^27^ The DHS samples are stratified by geographic region, and by urban/rural areas within each region. Since the early 2000s, the DHS has also provided global positioning data (GPS) for each PSU.^25^ A PSU is defined as a city block in an urban area and a village in a rural area. To anonymise the respondents, GPS coordinates are randomly displaced by the DHS Programme Team. Urban areas are randomly displaced up to 2 km, and rural areas are randomly displaced up to 5 km, with a further 1% displaced up to 10 m.^25,28^ Further details about the DHS can be found elsewhere.^25–27,29^

For this analysis, we included all births and deaths occurring within the first 28 days of postnatal life for births within the 5 years preceding the survey in each included country.^30^

### Meteorological data

We obtained ERA5-Land 2-metre air temperature and 2-metre dewpoint temperature from the Copernicus Climate Change Service of the European Commission, the European Centre for Medium-Range Weather Forecasts.^31^ ERA5-Land is a global gridded reanalysis dataset with a consistently high spatial (0.1°×0.1°) and temporal (1 hourly) resolution.^31^ It is considered highly reliable for capturing temperature trends and variations, demonstrating strong correlation coefficients (0.978–0.998) with observed meteorological data.^32,33^

### Exposure and outcomes

Daily mean temperature was constructed from hourly temperature values (calculated as the arithmetic mean of 24 hourly ERA5-Land 2-metre air temperature values for each calendar day) and used as the main exposure. We adjusted for dewpoint depression as a measure of humidity, defined as the difference between daily mean temperature and daily mean dewpoint temperature. Our outcome variables were (1) neonatal mortality (defined as death occurring from day 0 to 27); (2) very early neonatal mortality (defined as death occurring on day 0 only); (3) early neonatal mortality (defined as death occurring from day 1 to 6) and (4) late neonatal mortality (defined as death occurring from day 7 to day 27).

### Study design

We employed a time-stratified case-crossover design, which is used widely to assess acute health effects in relation to short-term environmental exposures, such as temperature or air pollution.^34–39^ The design compares each case’s exposure during the event period to their exposure during control periods when the health event did not occur.^40^ Control days were defined as the same day-of-week within the same month and year, resulting in 3–4 control days per case.^41^ Specifically, the event period is the day of neonatal death (day 0), and control periods are all other days falling on the same day of the week within the same calendar month and year as the event day. For example, if a death occurred on a Wednesday in March 2018, the control days would be all other Wednesdays in March 2018. Thus, by design, this approach adjusts for potential confounding by season and day-of-week^41–45^ as well as individual-level confounders, such as maternal age, education, socioeconomic status, healthcare and other sociodemographic characteristics that are unlikely to change within the small-time windows.^35,36,42^

### Statistical analysis

DHS and GPS data were linked using the PSU (identification number) ID. These data were then geospatially linked with the exposure data using the GPS coordinates and the date of neonatal mortality. We only linked data for which all GPS coordinates were available, excluding 165 deaths without linkable GPS coordinates.

We used conditional logistic regression, with the PSU and DHS Strata as the ID stratum (where each stratum is defined by the combination of the individual neonatal death case and the DHS stratum corresponding to a geographic region×urban/rural classification; the PSU ID and DHS stratum serve jointly as the stratification variable, meaning each case is compared only with its own control days within the same stratum),^42^ to obtain the OR of neonatal mortality at different temperature percentiles compared with the median temperature. We included the DHS strata in our analysis to account for PSU displacement of the GPS coordinates and the lack of GPS data on the place of death. Because the DHS is stratified by geographic regions and by urban/rural within each region, we hypothesised that any mortality, whether at home or hospital, was most likely to occur within the strata or region.

We modelled the nonlinear and lagged associations between temperature and neonatal mortality using distributed lag nonlinear models using natural cubic splines and linear terms.^46,47^ For each country, we fitted several models with linear and spline terms (varying df and knot placements) in the exposure and lag dimensions. A consistent 0–6 day lag structure was used across all outcome periods for comparability. For late neonatal deaths (days 7–27), this captures the acute temperature exposure in the week preceding death, which is the relevant window for assessing whether short-term temperature fluctuations trigger or exacerbate the medical conditions (infections, respiratory distress) that are the primary causes of late neonatal death. We used the lowest Akaike information criterion (AIC) value and examined residuals to determine the best-fitting models. For all countries, the best-fitting model used a non-linear term for temperature and a natural cubic spline for the lagged effects with 2 df. All models were adjusted for dewpoint depression. We did not adjust for air pollution due to a lack of reliable data in our study settings. Country-level estimates were then pooled using fixed effects, due to the consistently low between-country heterogeneity,^48,49^ to assess the overall association with temperature across all countries.

As sensitivity analyses, we conducted: (1) random-effects meta-analyses (Restricted Maximum Likelihood (REML) estimator); (2) analyses using 1st/99th temperature percentiles versus the median; and (3) minimum mortality temperature (MMT) centring, in which the exposure–response relationship was estimated using the AIC-best non-linear crossbasis specification. The MMT was identified empirically, and predictions were re-centred at this value (results in supplementary materials ST 15–ST 18). The fixed-effects pooled estimate is presented as primary but should be interpreted as a weighted average under the assumption of a common underlying effect; country-level findings are the primary results of this study.

Finally, the analyses were stratified by wealth group (low-, middle- and high-income) to assess whether the effect of ambient temperature varied by socioeconomic status. The wealth groups were generated from the DHS quintile groups, with the low-income group generated from the poorest and poor quintiles, middle-income group from the middle quintile, and the high-income group from the richest and richer quintiles.

### Patient and public involvement

This study had no public or patient involvement, and all the data used in the analysis are anonymised secondary data.

## Results

There were a total of 1538 neonatal deaths in the five East African countries recorded in the DHS data sets between 2011 and 2022, with 617 (40.1%) very early, 628 (40.8%) early and 293 (19.1%) late neonatal deaths. After removing cases with missing PSU and GPS data, the total number of neonatal deaths was 1373.

As shown in table 1, over 80% of all neonatal deaths occurred within the first week of life; 40.1% of which occurred on day 0. In Rwanda, over 50% of all neonatal deaths occurred within the first 24 hours after birth. Over the study period, the median temperature was 22.9°C (IQR 20.8°C–25.2°C) with a 5th percentile of 17.6°C and a 95th percentile temperature of 28.5°C. Kenya had the highest median temperature during the surveys at 25.2°C (IQR 21.7°C–27.8°C) followed by Tanzania and Uganda at 22.6°C (20.7°C–24.5°C) and 22.0°C (20.4°C–23.6°C), respectively. Rwanda had the lowest median temperature at 19.8°C (18.1°C–21.2°C) followed by Burundi at 20.6°C (19.1°C–22.3°C). Additional details shown in online supplemental material 1.

**Table 1:** Summary statistics of neonatal mortality and mean daily temperature

Country	Total N	Early neonatal deaths		Late neonatal deaths	Temperature, °C		
		Very early (day 0) N (%)	Early (day 1–6) N (%)	Late (day 7–27) N (%)	5th percentile	Median	95th percentile
Burundi	279	76 (27.2)	125 (44.8)	78 (28.0)	16.7	20.6	25.0
Kenya	437	179 (41.0)	184 (42.1)	74 (16.9)	17.2	25.2	30.4
Rwanda	153	81 (52.9)	42 (27.5)	30 (19.6)	16.1	19.8	22.8
Tanzania	261	97 (37.2)	117 (44.8)	47 (18.0)	17.7	22.6	27.2
Uganda	408	184 (45.1)	160 (39.2)	64 (15.7)	17.1	22.0	26.8
Total	1538	617 (40.1)	628 (40.8)	293 (19.1)	17.6	22.9	28.5

Values in parentheses are percentages of total neonatal deaths within the country.

N, number.

**Figure 1:**
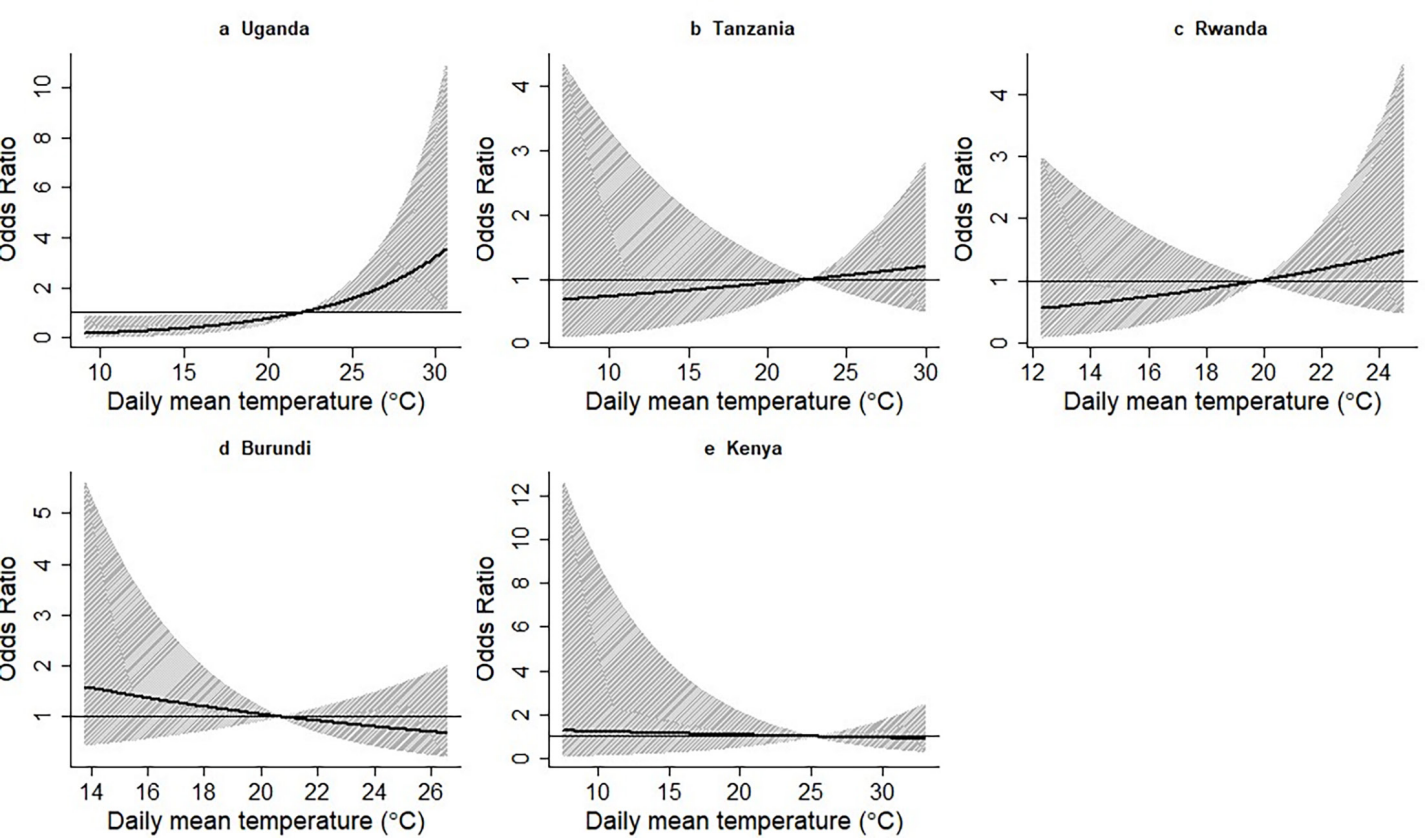
Association between mean daily temperature and the overall neonatal mortality.

Figure 1 shows the association between daily mean temperature exposures 0–6 days before death and the odds of neonatal mortality (0–27 days postpartum), centred at the median temperature. Overall, we observed between-country heterogeneity in the exposure–response associations. In Uganda, lower temperatures were associated with decreased odds of neonatal mortality, while higher temperatures were associated with increased odds (figure 1a). There was no strong evidence of association in other countries, although there was a tendency towards heterogenous effects. Tanzania and Rwanda (figure 1b,c) showed a tendency towards increased odds of mortality with increasing temperatures, while we observed the opposite in Burundi and Kenya (figure 1d,e). Exposure–response curves for very early, early and late neonatal mortality are provided in online supplemental materials 2–4.

**Figure 2:**
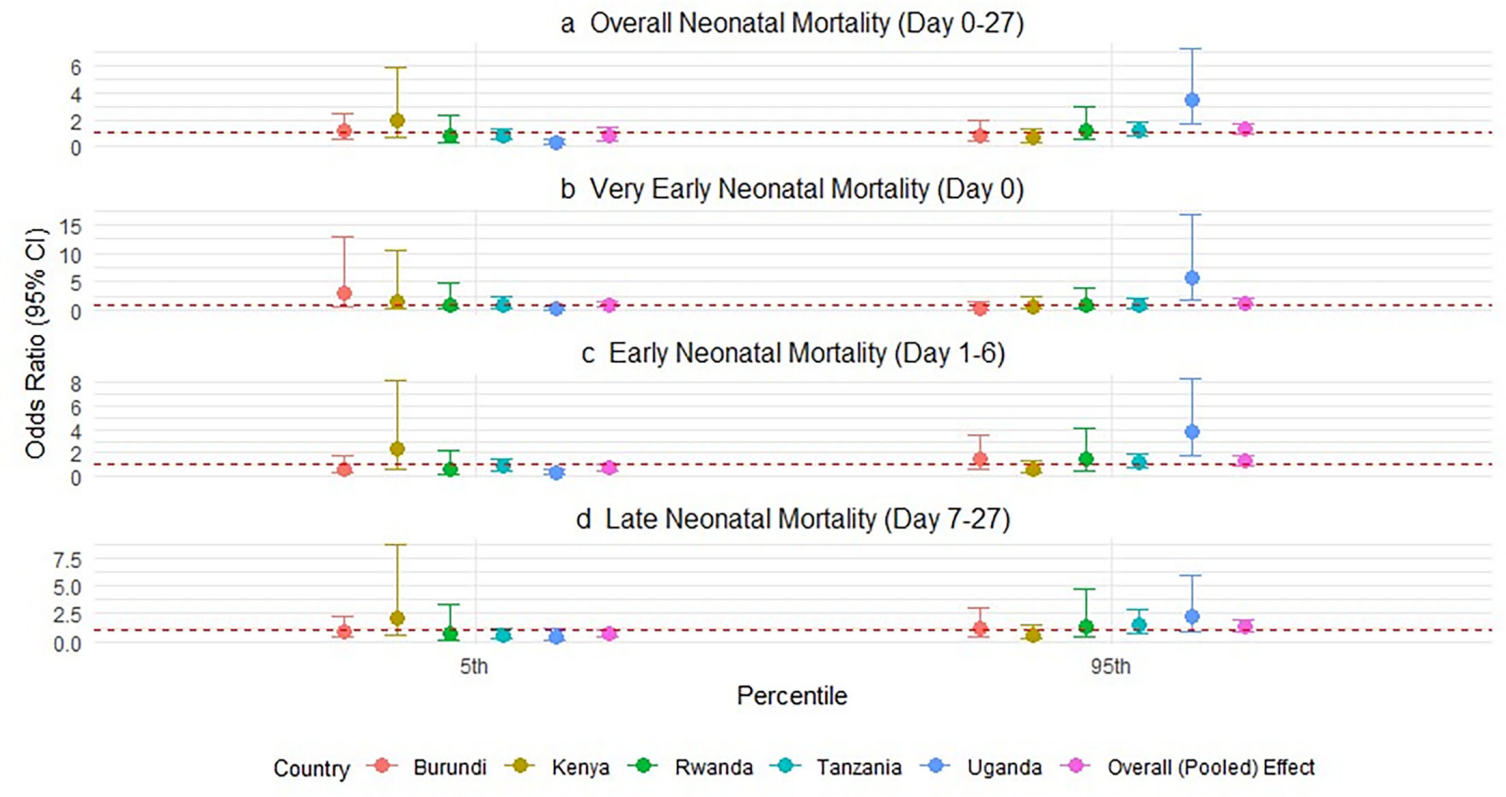
Association between 5th and 95th percentile temperatures compared with median temperature for country-level overall, very early, early and late neonatal mortality.

Figure 2 shows the ORs of overall, very early, early and late neonatal mortality with exposure to the 95th and 5th (vs the 50th) temperature percentiles. Overall, there was moderate between-country heterogeneity for the effect of temperature on neonatal mortality, with an overall trend towards increased mortality with exposure to high versus moderate temperatures (95th vs 50th percentiles) and decreased mortality with exposure to low versus moderate temperatures (5th vs 50th percentile). This trend was particularly notable in Uganda. For other countries, effect estimates were imprecise (ie, wide CIs crossing the null value of OR=1). For most country-outcome combinations outside of Uganda, the study was likely underpowered to detect an effect.

In pooled analysis, the odds of neonatal mortality increased by 27% (OR=1.27; 95% CI 0.95 to 1.70) with exposure to the 95th (vs 50th) temperature percentile, with moderate heterogeneity (I²=66.0%), and decreased by 22% (OR=0.78; 0.42 to 1.43) with exposure to the 5th (vs 50th) percentile, also with moderate heterogeneity (I²=64.0%). For very early neonatal mortality (figure 2b), the OR at the 95th percentile was 1.23 (0.75 to 2.03, I²=65.2%) and at the 5th percentile was 0.78 (0.45 to 1.35, I²=63.8%). For early neonatal mortality (figure 2c), the OR at the 95th percentile was 1.33 (0.96–1.84, I²=65.6%) and at the 5th percentile was 0.69 (0.48 to 1.00, I²=61.5%). The OR for late neonatal mortality (figure 2d) was 1.29 (0.87 to 1.92) at the 95th percentile, with negligible heterogeneity (I²=4.9%), and 0.73 (0.47 to 1.11) at the 5th percentile, also with negligible heterogeneity (I²=0.0%). In Uganda, a statistically significant association was observed between high environmental temperature and overall neonatal mortality (OR=3.54; 95% CI 1.73 to 7.28; figure 2a). Similar associations were observed with very early neonatal mortality at the 5th percentile (5.65; 1.89 to 16.69; figure 2b) and early neonatal mortality at the 95th percentile (3.75; 1.70 to 8.28; figure 2c). Detailed forest plots are provided in online supplemental materials 5–12.

**Figure 3:**
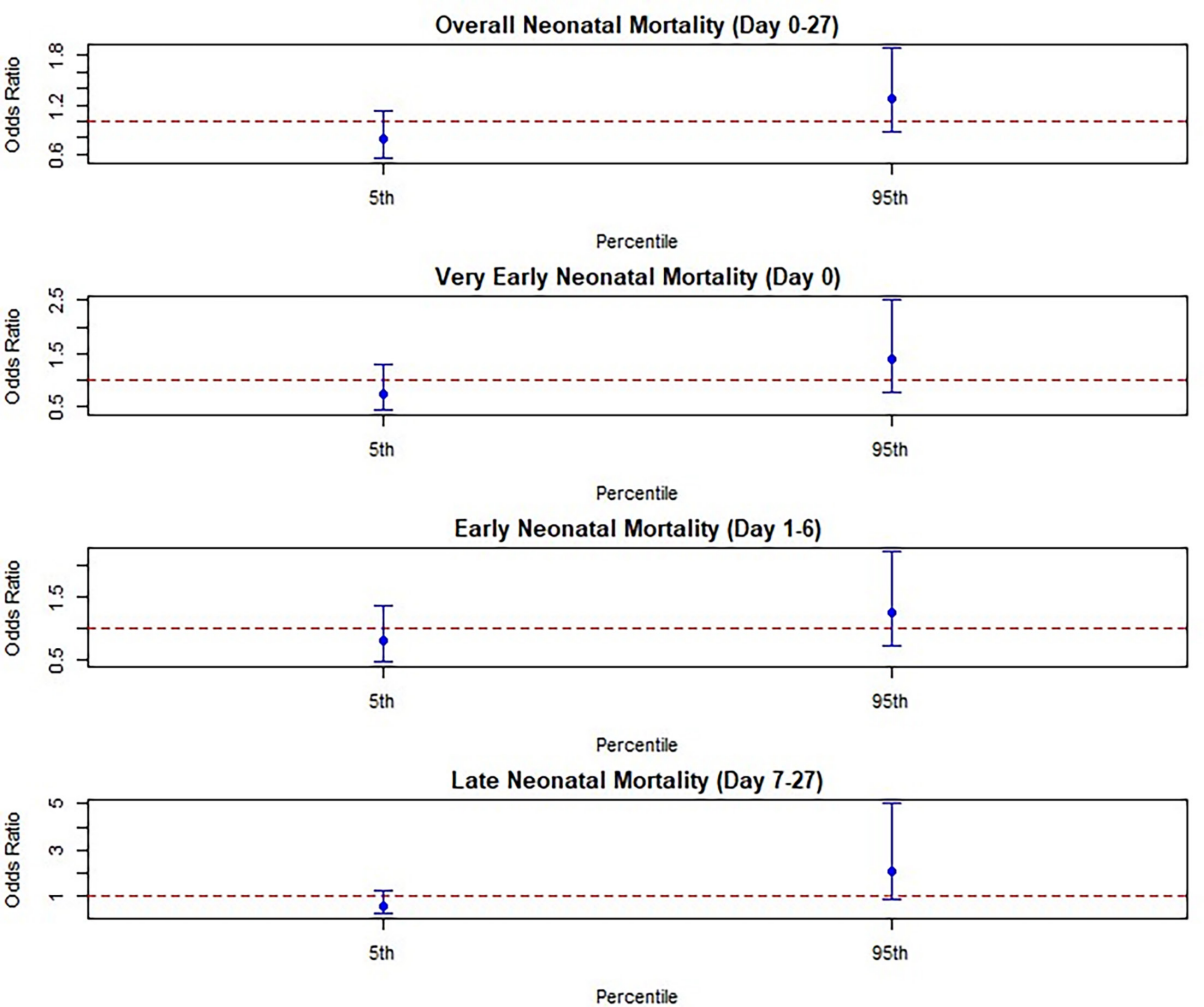
Association between 5th and 95th percentile temperatures compared with median temperature for pooled overall, very early, early and late neonatal mortality.

Figure 3 shows the ORs of the pooled overall, very early, early and late neonatal mortality with daily mean temperatures over the study period. Overall, there was moderate between-country heterogeneity for the effect of temperature on neonatal mortality with an overall trend towards increased mortality with high temperatures and decreased mortality with low temperatures, although results were imprecise. Detailed forest plots for each outcome are provided in online supplemental materials 13 and 14.

### Subgroup analysis

**Figure 4:**
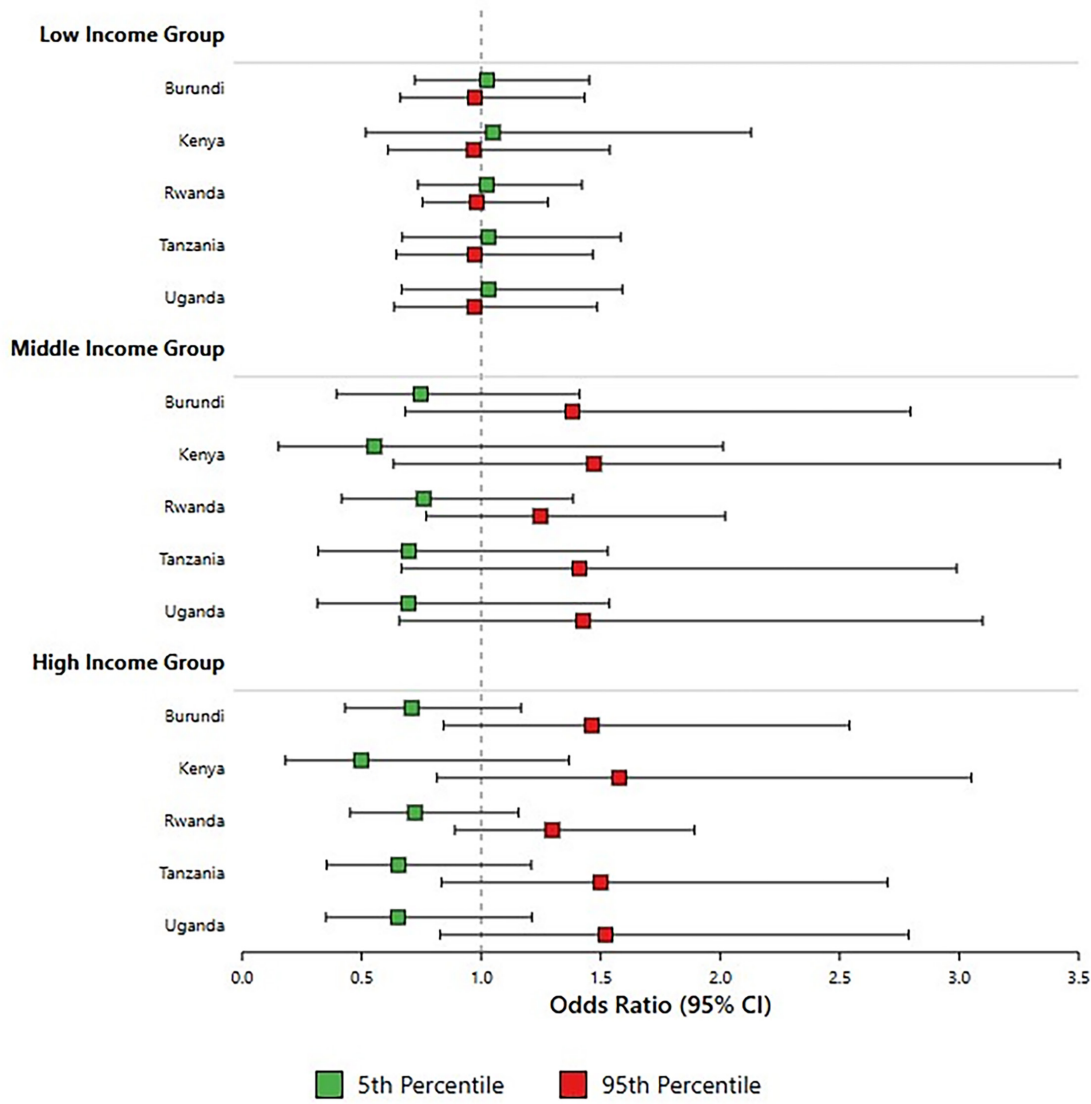
Subgroup analysis by income group.

The subgroup analysis results were imprecise for all the tertiles, as shown in figure 4. There was a total of 660 neonatal deaths in the low-income group, and 252 each in the middle- and high-income groups. However, for the middle- and high-income groups, exposure to the 95th and 5th temperature percentiles was invariably associated with increased and decreased odds of mortality, respectively, although effect estimates were imprecise due to small sample size. The effects of temperature were much more attenuated for both the 95th and 5th percentiles among the low-income group.

### Sensitivity analysis results

Sensitivity analyses using alternative percentile thresholds and centring values broadly supported our primary findings. At the 1st and 99th percentiles (vs the median), the direction of associations was consistent with the 5th/95th percentile analysis: the pooled OR at the 1st percentile for overall neonatal mortality was 0.66 (95% CI 0.35 to 1.26, I²=47.3%), and at the 99th percentile was 1.39 (0.81 to 2.39, I²=51.1%), with wider CIs reflecting fewer observations at the temperature extremes. Uganda’s strong heat association persisted at the 99th percentile (OR=4.23, 95% CI 1.64 to 10.86). For the MMT-centred analysis, the empirically estimated MMT frequently fell outside the 5th–95th percentile range (eg, 7.1°C for Tanzania, 9.1°C for Uganda), producing unstable estimates with extremely wide CIs. This instability reflects the difficulty of reliably estimating non-linear exposure–response curves—and hence the MMT—from the relatively small sample sizes available in each country. These findings support our primary approach of centring on the median temperature, which lies within the well-observed range of the data. Full sensitivity results are reported in online supplemental materials 15–18. Sensitivity analyses for the random-effects meta-analysis are presented in online supplemental material 15, showing very similar estimates to the fixed-effects pooled estimates. Online supplemental material 16 shows the summary for 1st and 99th temperature percentiles compared with the median, while online supplemental materials 17 and 18 are the MMT.

## Discussion

Overall, we found that higher temperatures were associated with increased odds of mortality, while lower temperatures were associated with decreased odds of mortality among the overall, very early (day 0), early (days 1–6) and late (days 7–27) neonatal deaths. However, strong effects were only observed in Uganda, and results were imprecise for the other four countries. In Uganda, higher temperatures were associated with increased odds of overall, very early and early neonatal mortality, while lower temperatures were associated with decreased mortality in these groups. Imprecise results were observed for late neonatal mortalities. There was no evidence of effect modification by the wealth groups. The attenuation of temperature effects among the low-income group may reflect the presence of competing risk factors (malnutrition, infections, limited skilled birth attendance) that dominate the risk profile, causing the relatively marginal effect of temperature to be less detectable. Differences in housing characteristics and care-seeking behaviours may also play a role; higher-income families may be more likely to deliver in enclosed health facilities that could trap heat, while lower-income families may deliver in more naturally ventilated settings.

The increased and decreased odds of mortality associated with higher and lower temperatures, respectively, for overall, very early and early neonatal mortalities in Uganda are consistent with other studies that have explored the relationship between temperature and neonatal mortality.^5,16,17,22^ The pathophysiological mechanisms through which higher temperatures lead to increased risk of neonatal mortality are still unknown. Suboptimal temperatures, both hot and cold, can lead to neonatal mortality through a variety of direct and indirect mechanisms. Direct mechanisms primarily involve the neonate’s immature thermoregulatory system.^6,13,50,51^ Neonates, especially preterm and low birth weight infants, have a narrower optimal body temperature range than adults.^5,6^ Their higher metabolic rate and lower sweating rate limit their ability to dissipate heat effectively,^5^ making them vulnerable to hyperthermia. Conversely, their high surface-area-to-mass ratio increases the risk of rapid heat loss and hypothermia.^5,16^

High ambient temperatures have been linked to preterm birth, placental abruption and gestational hypertension, all of which increase the risk of overall neonatal mortality.^3,5,50,52^ Neonatal hyperthermia can lead to lethargy, apnoea, dehydration, tachycardia and neurological complications,^13,14^ while signs of neonatal overheating may be clinically indistinguishable from sepsis, potentially leading to misdiagnosis and delayed appropriate management. However, there may be different medical causes according to the age of the newborns.^5^ Extreme temperature exposure has been linked with prematurity, low birth weight, maternal dehydration and other childbirth complications, which increase the risk of very early neonatal mortality.^3,6,16,21,22^ On the other hand, late neonatal mortality may be due to severe infections such as sepsis and pneumonia, which are often associated with hypothermia.^53–55^

Health systems in low-resource settings may be ill-equipped to manage the increased burden of neonatal complications arising from temperature extremes. Lack of access to essential equipment like incubators and infant warmers, as well as unreliable power supplies, can exacerbate the risks of both hypothermia and hyperthermia.^5^ Beyond the immediate physiological impacts, suboptimal temperatures can indirectly influence neonatal mortality by exacerbating existing environmental and socioeconomic vulnerabilities. Heat can negatively impact healthcare system responsiveness by contributing to healthcare worker exhaustion and disrupting agricultural production, ultimately affecting nutrition and access to care.^50^ Moreover, heatwaves can directly impede access to essential healthcare services, leading to delays in care-seeking and potentially fatal consequences for vulnerable neonates.^56^ The broader impacts of climate change, including extreme weather events such as droughts and floods, further compound these risks. These events can damage critical infrastructure, displace populations and disrupt food supplies, leading to increased rates of malnutrition and infectious diseases in neonates.^56^

In our analysis, we noted between-country heterogeneity on the effect of temperature on neonatal mortality. These results are similar to findings by Brimicombe *et al* in which no statistically significant effect of the 95th percentile was detected compared with the median heat exposure in the East Africa region (OR=1.02, 95% CI 0.96 to 1.08).^22^ However, their study pooled the East African region countries and therefore, unlike our analysis, between-country results are not teased out. Uganda’s outlier status may be explained by several factors. First, Uganda’s climate is strongly influenced by Lake Victoria, which increases humidity, particularly along its northern shore; higher humidity impairs evaporative heat loss and amplifies the physiological impact of high temperatures on neonates. Second, Uganda contributed the second largest number of neonatal deaths (n=408), increasing statistical power relative to other countries, particularly Rwanda (n=153). Finally, Uganda’s 95th percentile temperature (26.8°C) combined with high humidity near Lake Victoria may create effective heat stress exceeding what temperature alone suggests. These findings are also similar to Dimitrova *et al* in that, while they showed that 4.3% of all neonatal mortalities across 29 LMICs were associated with non-optimal temperatures (hot or cold), the effects across Burundi, Rwanda, Tanzania and Uganda were not statistically significant.^5^ Furthermore, a scoping review examining the effect of elevated ambient temperature on maternal, fetal and neonatal outcomes found mixed effects, with increased, decreased or no heat-related risk of neonatal mortality.^17^ Similar findings were reported in the systematic review by Lakhoo *et al* which reported mixed findings on the effect of heat on neonatal mortality across different regions and climate zones.^16^

The apparent insufficient evidence of the association between non-optimal temperatures and neonatal mortality in the East African region could be explained by several factors. First, the 5th and 95th temperature percentiles, chosen in our study as the cold and hot temperatures, ranged from 16.1°C to 17.6°C and 22.8°C to 30.4°C, respectively, and did not differ considerably from the median temperature, which ranged from 19.8°C to 25.2 °C. Generally, the countries had narrow temperature ranges. Second, the number of cases (mortalities) in our dataset was small, which may have led to increased sampling errors and wider CIs.^57^ Moreover, the number of cases at the 95th and 5th percentiles was even lower. Third, there could be factors other than temperature, such as preterm births and birth asphyxia, responsible for neonatal mortality in the East African region,^22^ although these are also linked with extreme temperatures. Finally, healthcare practices such as care for the newborn, which emphasise thermal care including Kangaroo Mother Care (KMC), especially for low- and preterm births, may attenuate the effect of ambient temperature on neonatal mortality in these settings.^58,59^

Regarding the apparent ‘protective’ effect (below unity) at the 5th percentile (pooled OR=0.78), we caution against interpreting this as evidence that low temperatures are inherently ‘protective’. The lower tail in these settings corresponds to relatively mild temperatures (16.1°C–17.7°C) and is close to the centring value, limiting exposure contrast and potentially placing the 5th percentile near a minimum-risk portion of the curve. In addition, ambient temperatures are an imperfect proxy for neonate’s thermal comfort (indoor conditions, clothing/bedding and care practices), which may attenuate cold-related risks. Established thermal protection practices—including immediate drying, skin-to-skin contact and KMC—are widely promoted and practised in East Africa and may reduce vulnerability to cold stress (particularly among preterm or low birth weight infants).^60^ A Cochrane meta-analysis showed KMC reduced neonatal mortality by 40% compared with conventional care (Risk Ration (RR) 0.60, 95% CI 0.39 to 0.92),^61^ and a trial in Ugandan hospitals demonstrated its cost-effectiveness.^60^ Beletew *et al* reported a pooled neonatal hypothermia prevalence of 57.2% in East Africa, indicating that, while hypothermia remains common, awareness and responses to cold stress are well-integrated into care practice.^62^ Finally, although we adjusted for humidity using dewpoint depression, residual confounding by other time-varying factors cannot be excluded. In contrast, comparable community-level interventions for heat stress protection are less established in these settings.^58,59^

### Strengths and limitations

Our analysis has several strengths. First, by employing a time-stratified case-crossover design, we controlled for time-invariant confounders, minimising the risk of confounding. Second, we analysed data per country, rather than at the regional or global level, which often masks the differential effects of temperature at the regional and/or global level, including confounders that may vary between countries. While this does not completely disaggregate data by fine temperature zones, it provides much more detailed country-specific and between-country results. Finally, to the best of our knowledge, this is the first study to explore the effects of temperature on neonatal mortality at different time periods, namely very early, early and late neonatal mortality.

However, several limitations must also be considered. First, given the relative rarity of the outcome, the sample size, as reported above was very small, which may have affected the CIs. After excluding cases with missing GPS data, only 1373 deaths were available across five countries, and these were further subdivided by outcome period and country for the primary analyses. The number of observations at the extreme temperature percentiles (5th and 95th) was smaller still. Thus, the study may have been underpowered to detect modest temperature effects in country-specific and age-stratified analyses. Second, in the DHS data, the GPS coordinates are linked to the PSU. No information about the place of death is provided.^25^ Thus, the child could plausibly have died in an area with a different temperature than that assigned in our analysis. Thus, non-differential misclassification may have occurred, biasing results towards the null and potentially masking the true effects. However, we anticipate that mean daily temperatures will not vary greatly between the DHS strata.^27^ Third, for late neonatal mortality, the 0–6-day lag structure captures only acute temperature exposures preceding death and does not account for potential effects of temperature exposures around the time of birth, which would require a different analytical approach (eg, a cohort design). Consequently, our findings should be interpreted as estimates of acute temperature effects rather than the cumulative impact of thermal exposure across the neonatal period. Fourth, residual confounding cannot be excluded. While the time-stratified case-crossover design inherently controls for all stable individual-level characteristics and adjusts for seasonality and day-of-week effects by design, some time-varying factors may remain uncontrolled. These include air pollution, rainfall, infectious disease outbreaks, food insecurity, healthcare service disruptions and other environmental exposures that may vary alongside temperature and influence neonatal survival. Air pollution is particularly relevant because it is often correlated with temperature and has been independently associated with adverse neonatal outcomes. Fifth, our analysis used daily mean temperature as the primary exposure metric. While this captures overall thermal conditions, it may not adequately represent short-duration extreme heat exposures that could be particularly relevant for neonatal physiology. Use of maximum temperature, minimum temperature, apparent temperature or heat index measures may yield different estimates. Finally, our analysis may suffer from recall bias since the information about births and mortality is collected for the 5 years preceding the survey. This means that we may have included stillbirths as neonatal deaths and/or misclassified neonatal deaths by the period of death. This is likely to lead to non-differential misclassification which biases the true estimates towards the null. We tried to minimise this by focusing on the most recent survey.

## Conclusion and recommendation

While our overall results show heterogeneity and uncertainty, we note that higher temperatures were generally associated with increased odds of mortality. There is therefore a need to consider incorporating heat mitigation strategies into the care for newborn policies and practices, especially for the very early and early neonatal periods. Specific context-appropriate interventions may include: community education for mothers and caregivers on recognising signs of neonatal overheating; ensuring adequate ventilation in maternity wards and postnatal care areas; scheduling postnatal home visits during cooler parts of the day where feasible; promoting adequate breastfeeding for hydration during hot periods; and integrating heat-health alerts into existing early warning systems for maternal and newborn health.^55^ There is also a need to develop and implement country-specific and age-specific guidelines for thermal management of neonates, especially in Uganda.

In conclusion, temperature-related neonatal mortality risk varies across East African countries, with only Uganda showing significant associations with high and low temperatures. The effects of temperature on neonatal mortality may be more pronounced in the first 6 days of life, perhaps linked to childbirth complications. The findings also suggest that other factors may play a more significant role in neonatal mortality in East Africa. There is a need for further research to understand policies, practices and mechanisms underlying the relationship between temperature and neonatal mortality in Uganda. There is also a need to explore the effects of temperature on neonatal mortality in the different climate/temperature zones.

## Supplementary material

10.1136/bmjph-2025-004085 online supplemental file 1

10.1136/bmjph-2025-004085 online supplemental file 2

10.1136/bmjph-2025-004085 online supplemental file 3

10.1136/bmjph-2025-004085 online supplemental file 4

10.1136/bmjph-2025-004085 online supplemental file 5

10.1136/bmjph-2025-004085 online supplemental file 6

10.1136/bmjph-2025-004085 online supplemental file 7

10.1136/bmjph-2025-004085 online supplemental file 8

10.1136/bmjph-2025-004085 online supplemental file 9

10.1136/bmjph-2025-004085 online supplemental file 10

10.1136/bmjph-2025-004085 online supplemental file 11

10.1136/bmjph-2025-004085 online supplemental file 12

10.1136/bmjph-2025-004085 online supplemental file 13

10.1136/bmjph-2025-004085 online supplemental file 14

10.1136/bmjph-2025-004085 online supplemental file 15

10.1136/bmjph-2025-004085 online supplemental file 16

10.1136/bmjph-2025-004085 online supplemental file 17

10.1136/bmjph-2025-004085 online supplemental file 18

## Data Availability

Data are available in a public, open access repository.
