# Supplementary figures and images for: Ambient temperature extremes and neonatal mortality: a time-stratified case-crossover analysis using Demographic and Health Survey data from East Africa (2011–2022)
Source: BMJ Public Health. 2026 Jul 22;4(3):e004085. doi: 10.1136/bmjph-2025-004085 (PMC13404639; doi:10.1136/bmjph-2025-004085)

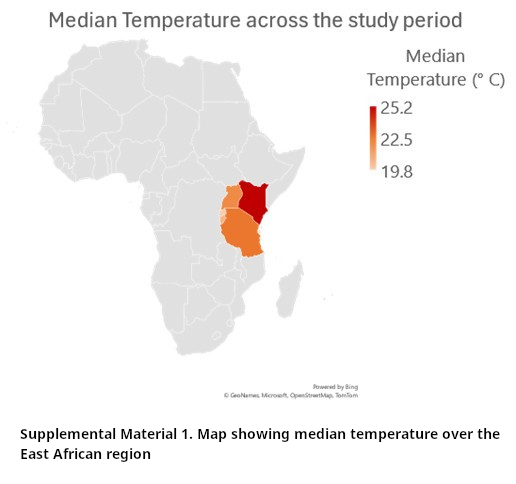

Supplement: online supplemental file 1 [file bmjph-4-3-s001.tiff]

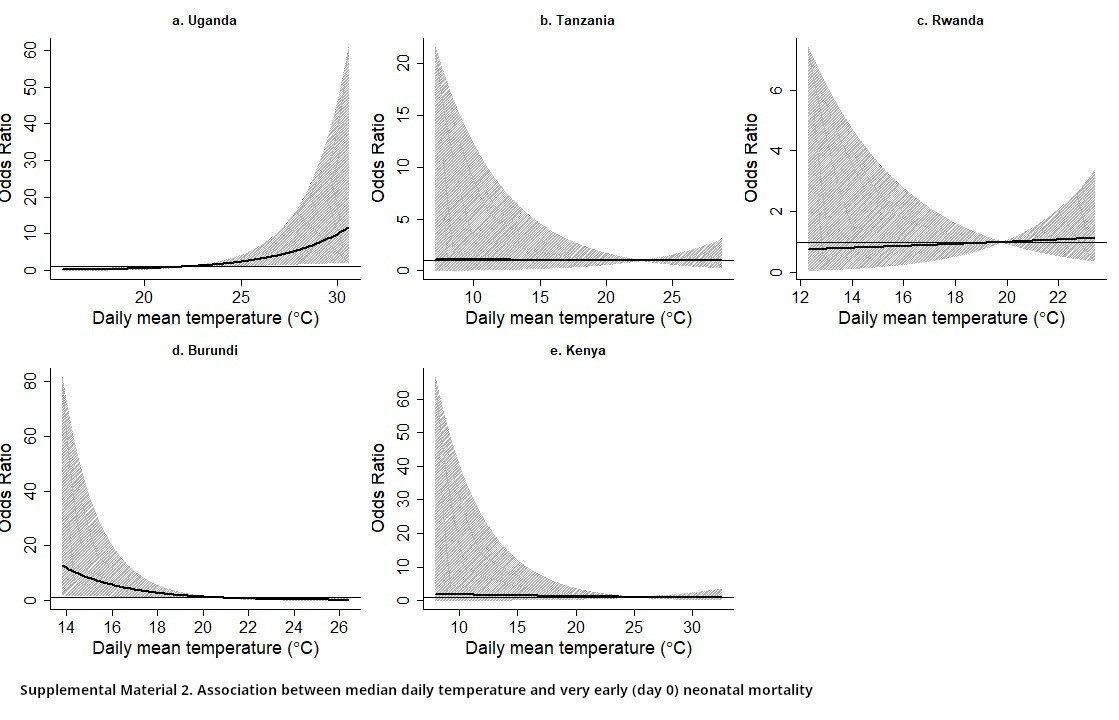

Supplement: online supplemental file 2 [file bmjph-4-3-s002.tiff]

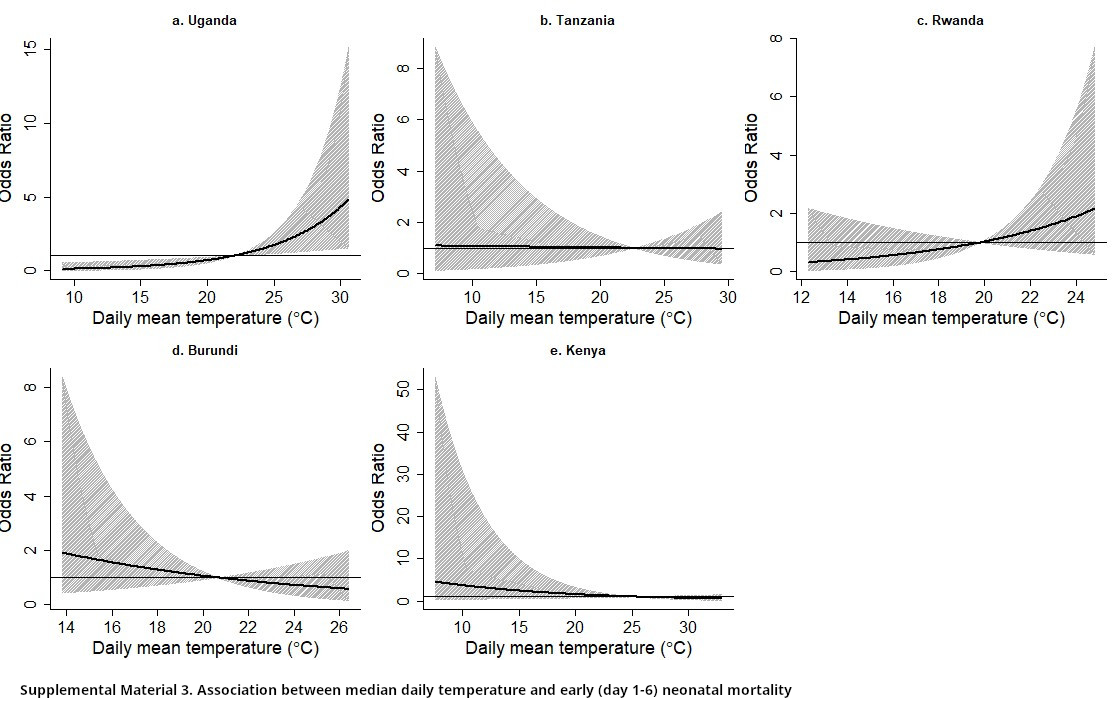

Supplement: online supplemental file 3 [file bmjph-4-3-s003.tiff]

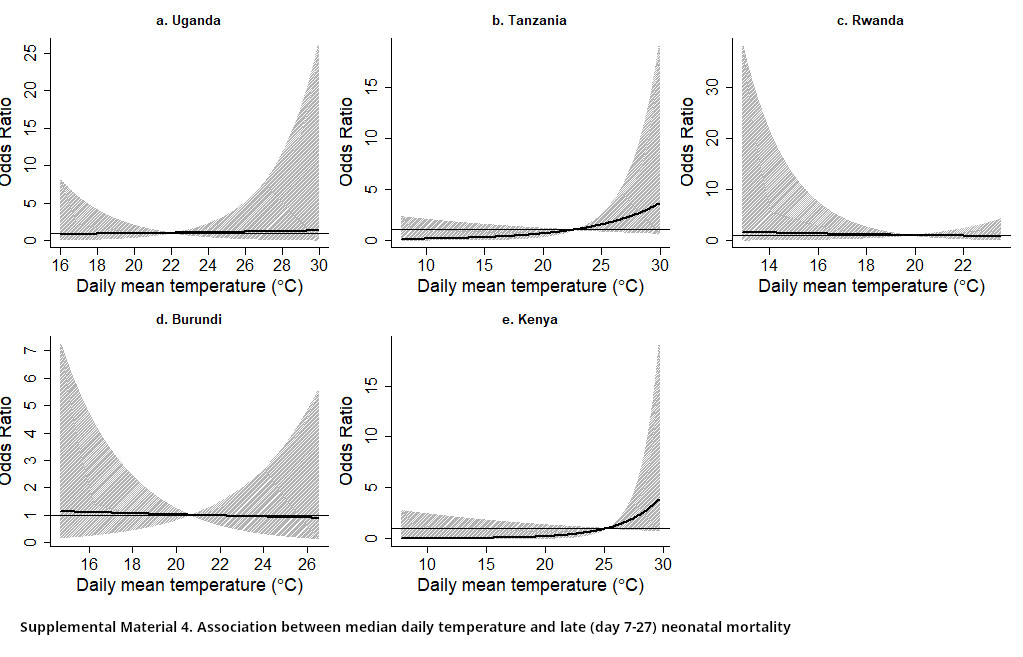

Supplement: online supplemental file 4 [file bmjph-4-3-s004.tiff]

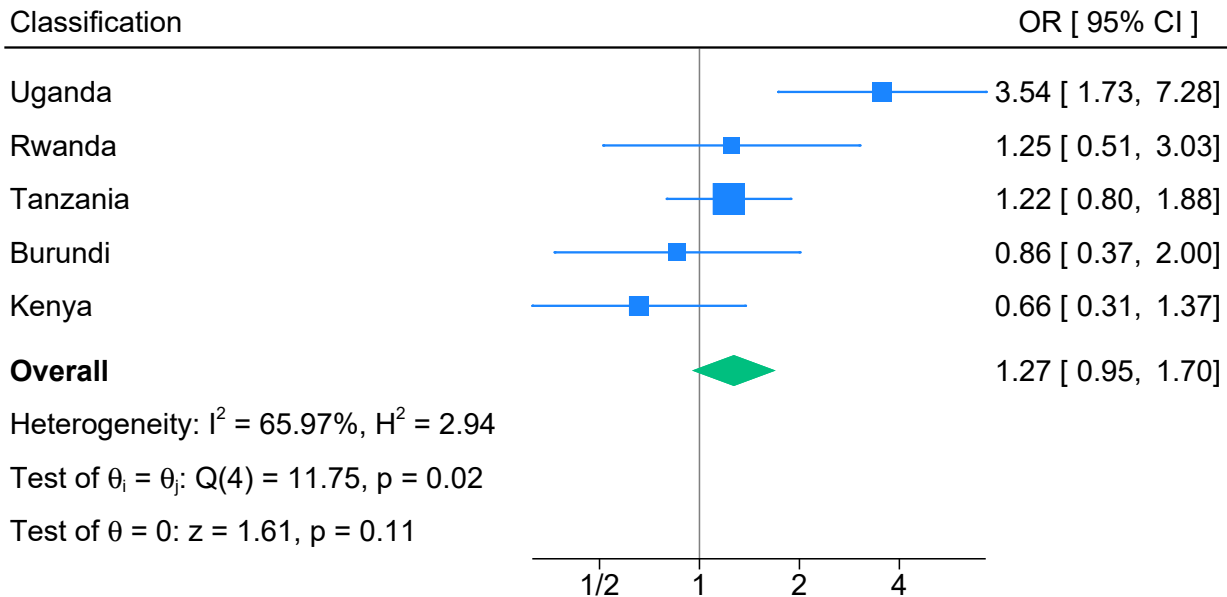

Supplement: online supplemental file 5 [file bmjph-4-3-s005.pdf]

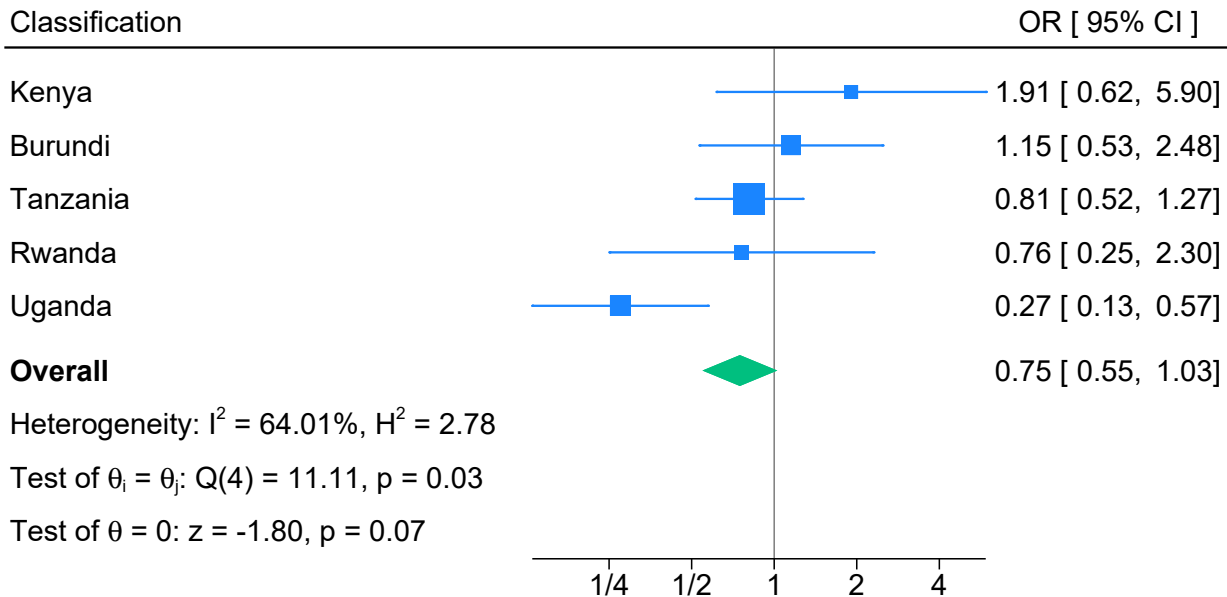

Supplemental Material 6. Overall neonatal mortality: Forest plot for 5th percentile vs median

Supplement: online supplemental file 6 [file bmjph-4-3-s006.pdf]

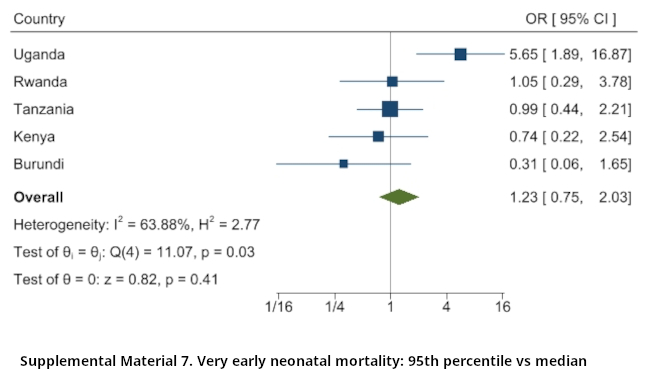

Supplement: online supplemental file 7 [file bmjph-4-3-s007.tiff]

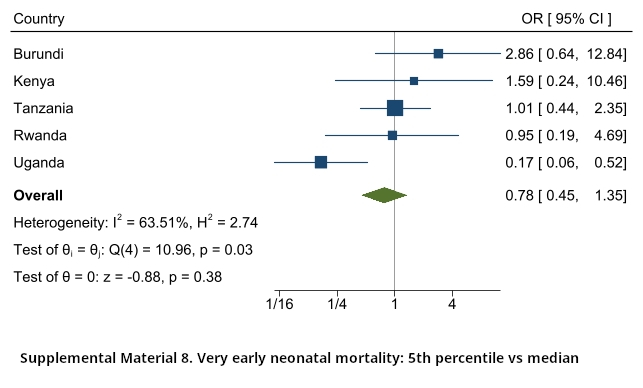

Supplement: online supplemental file 8 [file bmjph-4-3-s008.tiff]

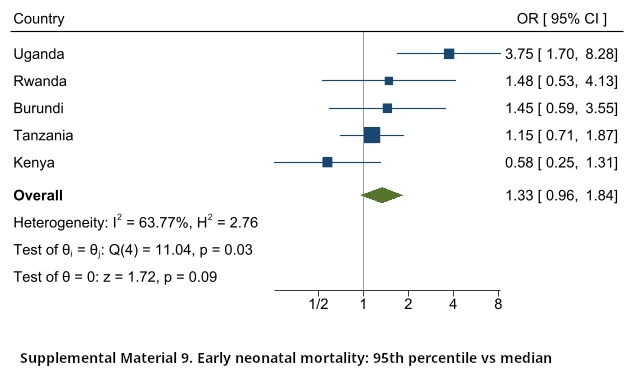

Supplement: online supplemental file 9 [file bmjph-4-3-s009.tiff]

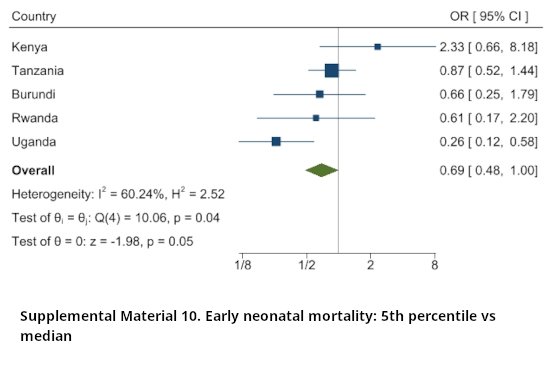

Supplement: online supplemental file 10 [file bmjph-4-3-s010.tiff]

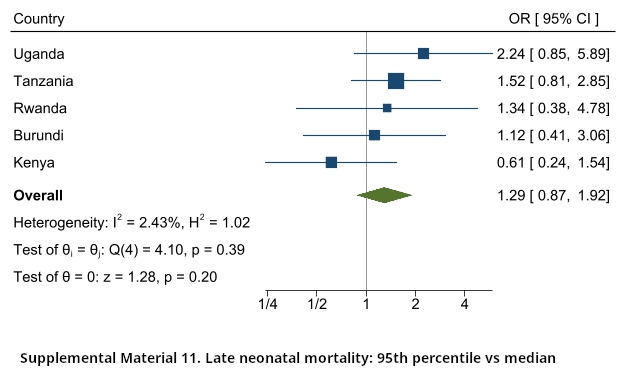

Supplement: online supplemental file 11 [file bmjph-4-3-s011.tiff]

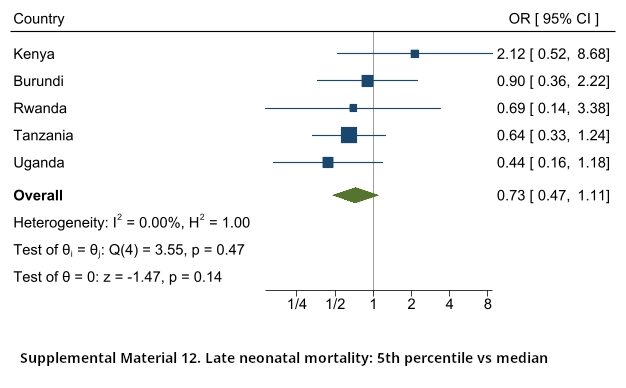

Supplement: online supplemental file 12 [file bmjph-4-3-s012.tiff]

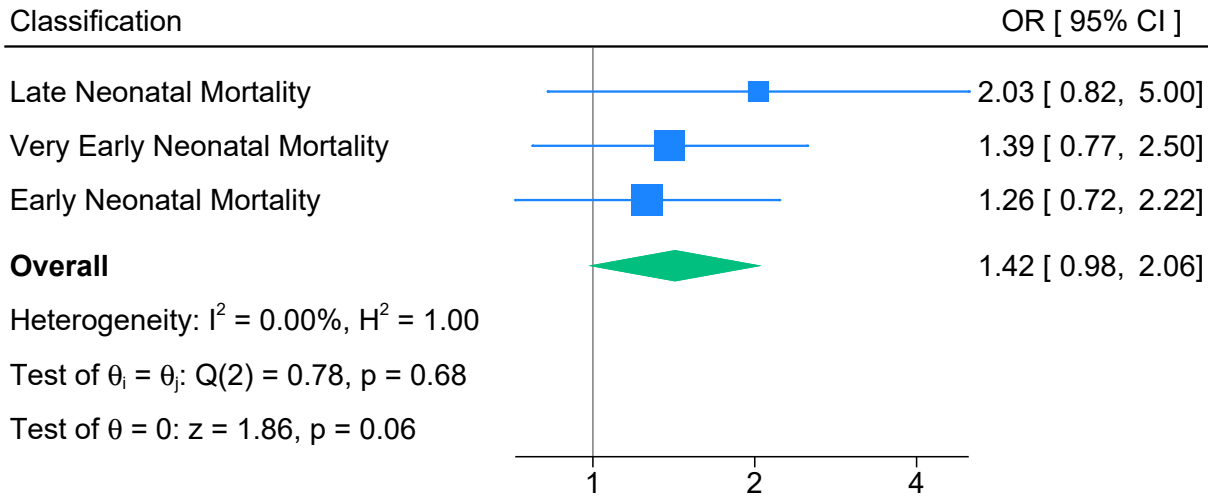

Supplement: online supplemental file 13 [file bmjph-4-3-s013.pdf]

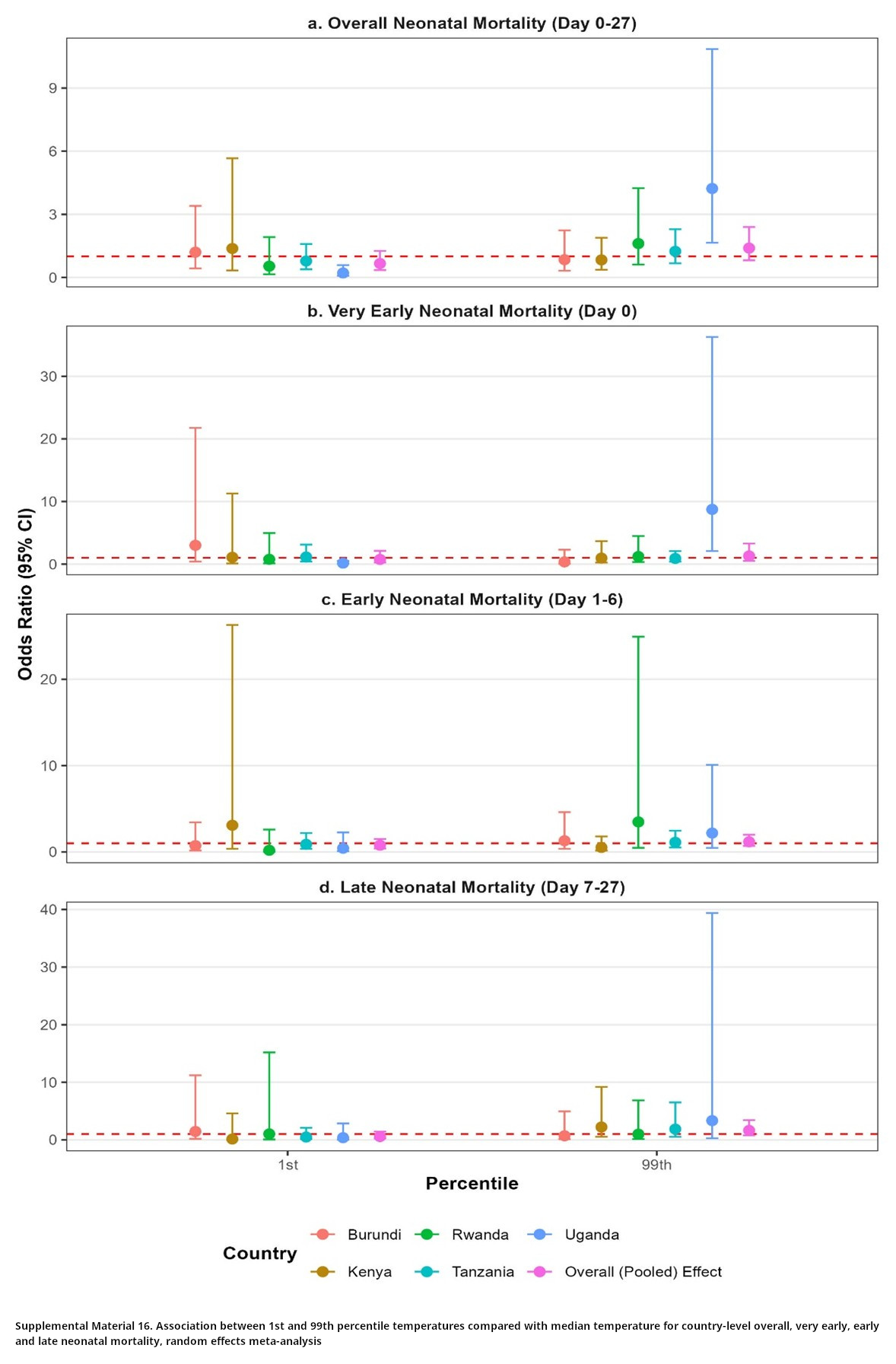

Supplement: online supplemental file 16 [file bmjph-4-3-s016.tiff]

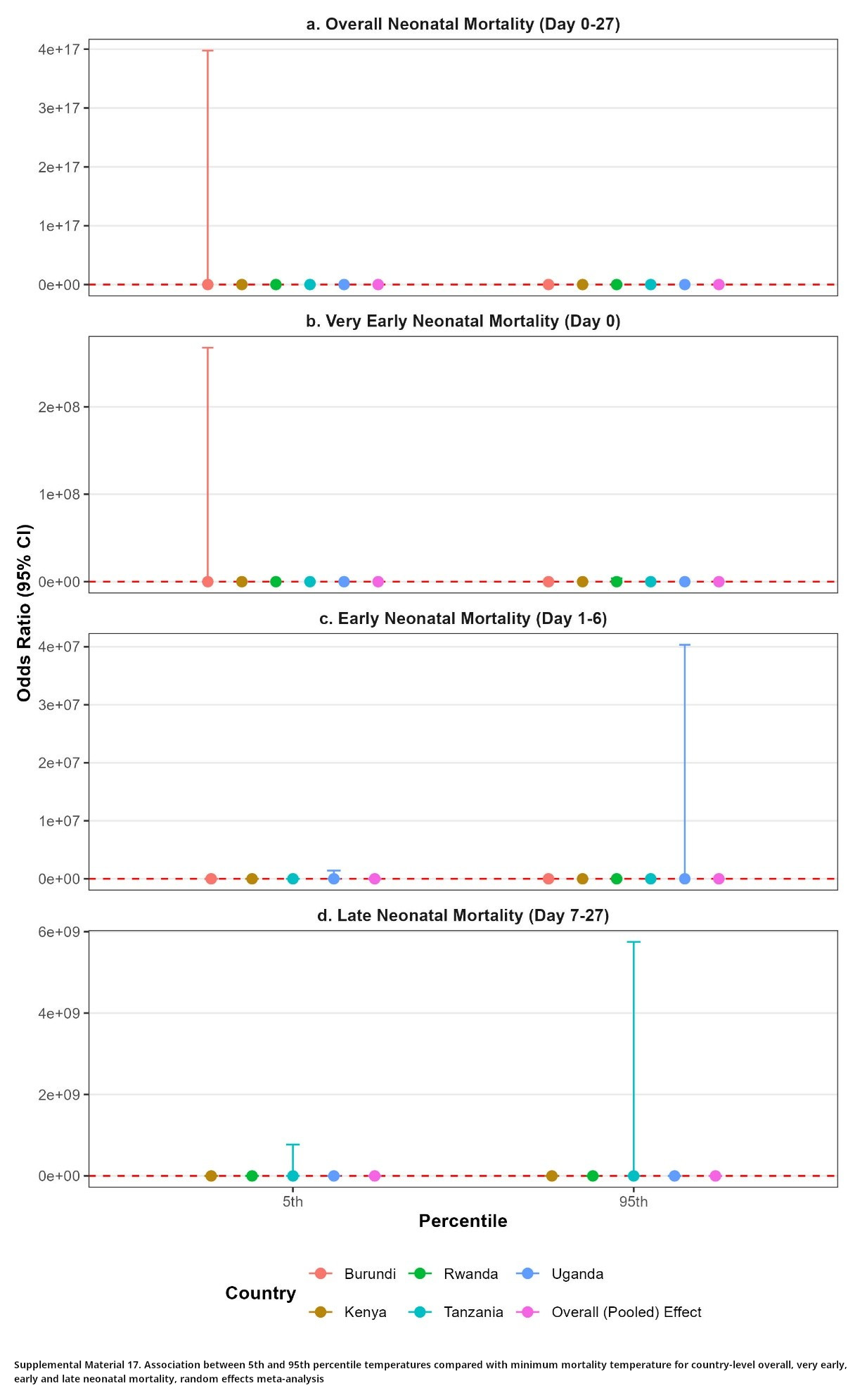

Supplement: online supplemental file 17 [file bmjph-4-3-s017.tiff]

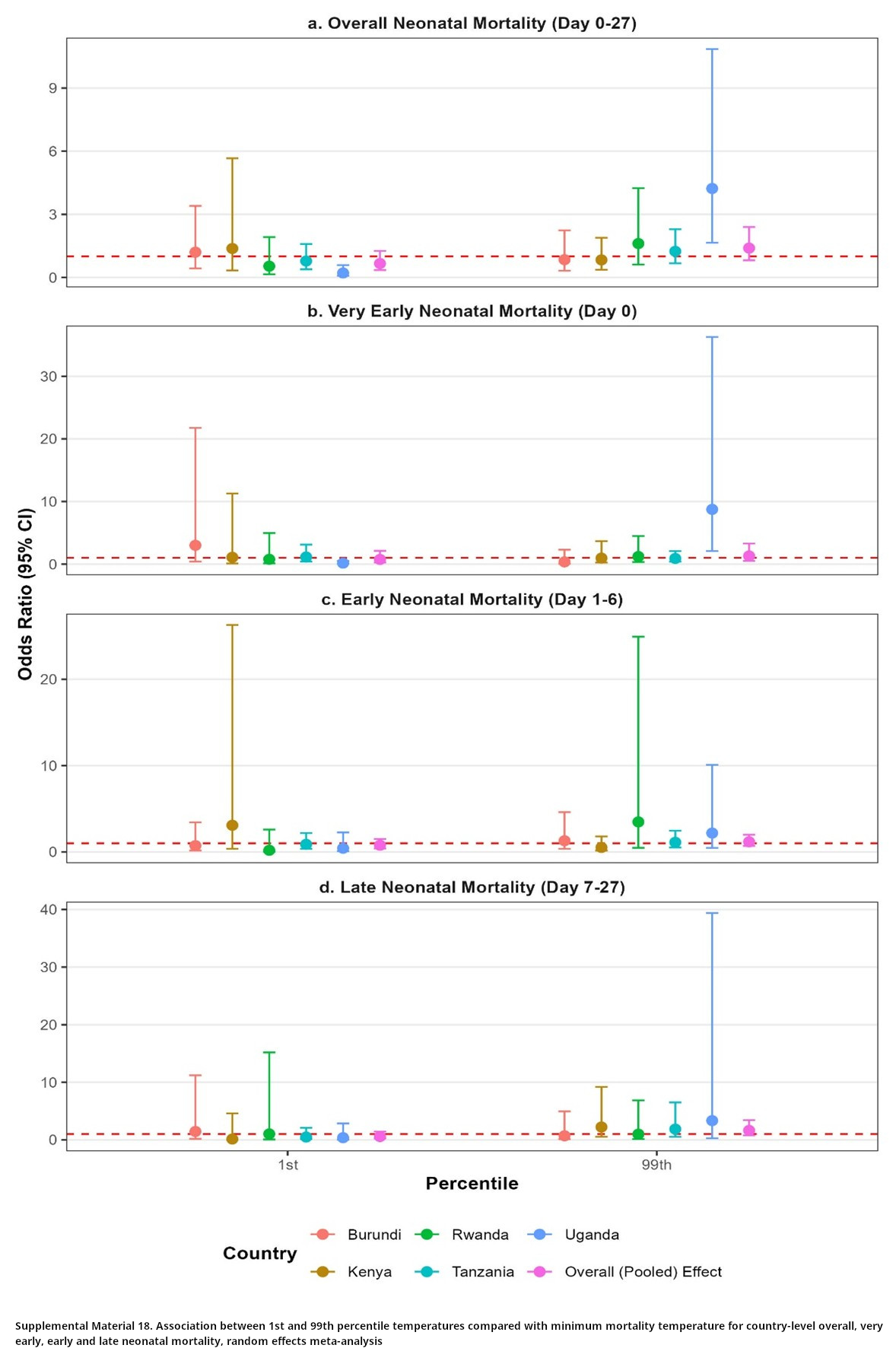

Supplement: online supplemental file 18 [file bmjph-4-3-s018.tiff]
