# Supplementary material for: Ambient temperature extremes and neonatal mortality: a time-stratified case-crossover analysis using Demographic and Health Survey data from East Africa (2011–2022)
Source: BMJ Public Health. 2026 Jul 22;4(3):e004085. doi: 10.1136/bmjph-2025-004085 (PMC13404639; doi:10.1136/bmjph-2025-004085)
Supplement: online supplemental file 15 [file bmjph-4-3-s015.pdf]

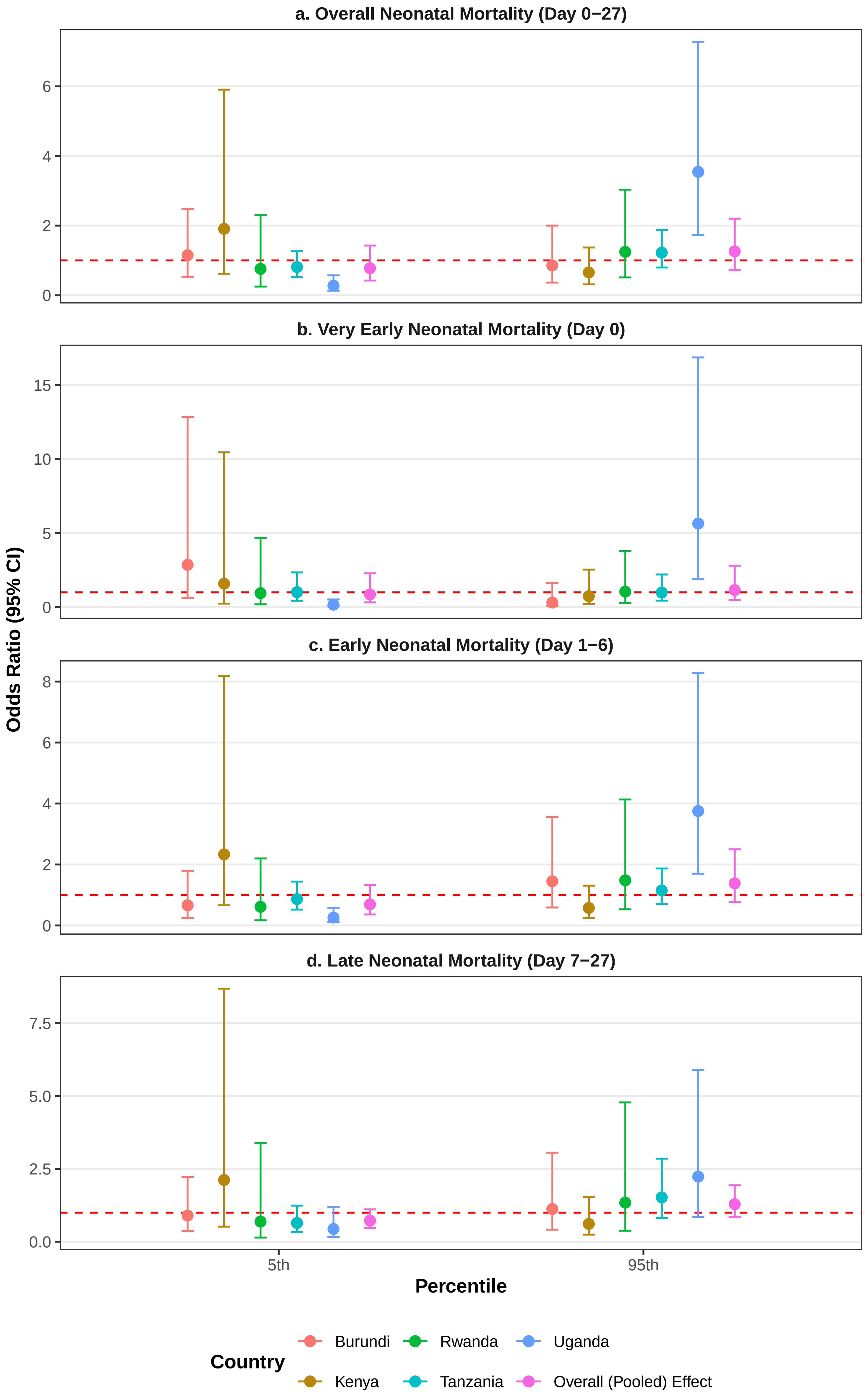

Supplemental Material 15. Association between 5th and 95th percentile temperatures compared with median
